# Association between Sleep Disturbances and Liver Status in Obese Subjects with Nonalcoholic Fatty Liver Disease: A Comparison with Healthy Controls

**DOI:** 10.3390/nu11020322

**Published:** 2019-02-02

**Authors:** Bertha Araceli Marin-Alejandre, Itziar Abete, Irene Cantero, Jose I. Riezu-Boj, Fermín I. Milagro, J. Ignacio Monreal, Mariana Elorz, José Ignacio Herrero, Alberto Benito-Boillos, Jorge Quiroga, Ana Martinez-Echeverria, Juan Isidro Uriz-Otano, María Pilar Huarte-Muniesa, Josep A. Tur, J. Alfredo Martínez, M. Angeles Zulet

**Affiliations:** 1 Department of Nutrition, Food Sciences and Physiology and Centre for Nutrition Research, Faculty of Pharmacy and Nutrition, University of Navarra, 31008 Pamplona, Spain; bmarin.1@alumni.unav.es (B.A.M.-A.); iabetego@unav.es (I.A.); icgonzalez@unav.es (I.C.); jiriezu@unav.es (J.I.R.-B.); fmilagro@unav.es (F.I.M.); mazulet@unav.es (M.A.Z.); 2 Biomedical Research Centre Network in Physiopathology of Obesity and Nutrition (CIBERobn), Instituto de Salud Carlos III, 28029 Madrid, Spain; pep.tur@uib.es; 3 Navarra Institute for Health Research (IdiSNA), 31008 Pamplona, Spain; jimonreal@unav.es (J.I.M.); marelorz@unav.es (M.E.); iherrero@unav.es (J.I.H.); albenitob@unav.es (A.B.-B.); jquiroga@unav.es (J.Q.); ana.martinez.echeverria@cfnavarra.es (A.M.-E.); jurizota@cfnavarra.es (J.I.U.-O.); phuartem@cfnavarra.es (M.P.H.-M.); 4 Clinical Chemistry Department, Clinica Universidad de Navarra 31008, Pamplona, Spain; 5 Department of Radiology, Clinica Universidad de Navarra 31008, Pamplona, Spain; 6 Liver Unit, Clinica Universidad de Navarra 31008, Pamplona, Spain; 7 Centro de Investigación Biomédica en Red de Enfermedades Hepáticas y Digestivas (CIBERehd), 28029 Madrid, Spain; 8 Department of Internal Medicine, Clinica Universidad de Navarra, 31008 Pamplona, Spain; 9 Department of Gastroenterology, Complejo Hospitalario de Navarra, 31008 Pamplona, Spain; 10 Research Group on Community Nutrition and Oxidative Stress, University of Balearic Islands, 07122 Palma, Spain; 11 Madrid Institute of Advanced Studies (IMDEA Food), 28049 Madrid, Spain

**Keywords:** Obesity, NAFLD, sleep, sleep duration, sleep disruption, Pittsburgh Sleep Quality Index

## Abstract

The relevance of sleep patterns in the onset or evolution of nonalcoholic fatty liver disease (NAFLD) is still poorly understood. Our aim was to investigate the association between sleep characteristics and hepatic status indicators in obese people with NAFLD compared to normal weight non-NAFLD controls. Ninety-four overweight or obese patients with NAFLD and 40 non-NAFLD normal weight controls assessed by abdominal ultrasonography were enrolled. Hepatic status evaluation considered liver stiffness determined by Acoustic Radiation Force Impulse elastography (ARFI) and transaminases. Additionally, anthropometric measurements, clinical characteristics, and biochemical profiles were determined. Sleep features were evaluated using the Pittsburgh Sleep Quality Index (PSQI). Hepatic status parameters, anthropometric measurements, and clinical and biochemical markers differed significantly in NAFLD subjects compared to controls, as well as sleep efficiency, sleep disturbance score, and sleep quality score. In the NAFLD group, a higher prevalence of short sleep duration (*p* = 0.005) and poor sleep quality (*p* = 0.041) were found. Multivariate-adjusted odds ratio (95% confidence interval) for NAFLD considering sleep disturbance was 1.59 (1.11–2.28). Regression models that included either sleep disturbance or sleep quality predicted up to 20.3% and 20.4% of the variability of liver stiffness, respectively, and after adjusting for potential confounders. Current findings suggest that sleep disruption may be contributing to the pathogenesis of NAFLD as well as the alteration of the liver may be affecting sleep patterns. Consequently, sleep characteristics may be added to the list of modifiable behaviors to consider in health promotion strategies and in the prevention and management of NAFLD.

## 1. Introduction

Nonalcoholic fatty liver disease (NAFLD) is a highly prevalent cause of hepatic disease around the world, which will putatively emerge as the most important cause of end-stage liver disease in the next decades [1]. NAFLD is described as the excessive accumulation of hepatic fat in the absence of history of alcohol abuse or other causes of secondary hepatic steatosis [2,3]. Multiple factors are involved in the complex pathogenesis of NAFLD, such as obesity, lipotoxicity, inflammation, unbalanced dietary intake, low physical activity, gut microbiota, and socioeconomic aspects, which might contribute to the development of this burden, along with genetic predisposition [1,4,5,6,7]. Currently, it is suggested that the link between NAFLD, metabolic syndrome and its individual components, including hypertension, type 2 diabetes mellitus, and cardiovascular disease, is more complex than previously believed and that NAFLD may be both a cause and a consequence of the metabolic syndrome [8]. Additionally, NAFLD can potentially lead from simple steatosis to the development of nonalcoholic steatohepatitis (NASH), a condition that often progresses to fibrosis, cirrhosis, and hepatocellular carcinoma, although these entities may often follow an asymptomatic course [1,9,10].

On the other hand, inadequate sleep has been associated with poor health outcomes [11], such as obesity [12], type 2 diabetes mellitus [12,13], cardiometabolic diseases [14], and all-cause mortality [11], as well as to the risk [15] and progression [16] of NAFLD. Sleep disruption has been reported to modify feeding behaviors and timing of food intake, to promote obesity, and to alter insulin sensitivity in adipose tissues in both humans and murine models [16,17,18]. Moreover, it is proposed that these metabolic alterations related to disrupted sleep patterns may be partially mediated by changes in gut microbiota [17,19]. Regarding the association of sleep duration and NAFLD, conflicting and often inconsistent results have been reported [15,20,21,22]. Thus, further investigation is needed to clarify this relationship.

Furthermore, specific characteristics concerning the sleep pattern analysis, such as time to fall asleep or daytime sleepiness, might have a relevant role in the onset and evolution of NAFLD [16]. However, it is unclear if sleep disruption is a cause or a consequence of the liver dysfunction in NAFLD patients.

Currently, the association of the characteristics of sleep patterns with the onset and progression of liver steatosis are still inconclusive and poorly understood. Moreover, the number of studies which investigate sleep features other than sleep duration remain scarce. Therefore, the aim of this study was to evaluate the relationships between sleep quality and hepatic status in obese patients with NAFLD compared to normal weight non-NAFLD controls considering sleep duration, sleep efficiency, sleep disturbance, and overall sleep quality scores.

## 2. Materials and Methods

### 2.1. Subjects

Overweight or obese (Body Mass Index (BMI) ≥ 25 kg/m^2^) participants with NAFLD were included in the study. A total of 40 healthy normal weight (BMI < 25.0 kg/m^2^) participants were also recruited as a control group. Patients with NAFLD were participants of the Fatty Liver in Obesity (FLiO) study (evaluated at baseline) who accurately completed the sleep questionnaires. The presence of hepatic steatosis was determined by abdominal ultrasonography (Siemens ACUSON S2000 and S3000) in the absence of other causes of liver disease reported by the subjects in a face to face clinical interview. These causes include excessive alcohol consumption, hepatitis C, parenteral nutrition, medications, and Wilson’s disease, among others [3]. The ultrasonography assessment consisted of a qualitative visual evaluation of liver echogenicity, measurements of the difference between the kidneys and the liver in the echo amplitude, and determination of the clarity of the structures of the intrahepatic vessels [23,24]. All the ultrasound examinations were performed and evaluated by the same qualified radiologist at the department of Ultrasonography and Radiology of the University of Navarra. Exclusion criteria included the following conditions: Known liver disease other than NAFLD, weight loss ≥ 3kg in the last 3 months, alcohol consumption > 21 and > 14 units of alcohol a week for men and women, respectively [25], endocrine disorders (hyperthyroidism or uncontrolled hypothyroidism), drug treatment with immunosuppressants, cytotoxic agents, systemic corticosteroids, or other drugs that could potentially cause hepatic steatosis or alteration of liver tests [3], presence of active autoimmune diseases or requiring pharmacological treatment, use of weight modifiers, and presence of severe psychiatric disorders. This information was declared by the subjects in the clinical interview before their enrollment in the study. All the procedures performed in the study were in accordance with the Declaration of Helsinki. Written informed consent was obtained from each individual prior to participation in the study, which was approved by the Research Ethics Committee of the University of Navarra (ref. 54/2015). The study protocol was properly registered in www.clinicaltrails.gov (FLiO: Fatty Liver in Obesity study; NCT03183193).

### 2.2. Anthropometric and Biochemical Measurements

Anthropometric measurements (body weight, height and waist circumference), body composition by dual-energy x-ray absorptiometry (DXA, Lunar iDXA, encore 14.5, Madison, WI, USA), and blood pressure (Intelli Sense. M6, OMRON Healthcare, Hoofddorp, The Netherlands) were determined in fasting conditions under previously described procedures [26] at the Metabolic Unit of the University of Navarra. Fasting blood samples were collected, processed (15 min; 3500 rpm; 5 °C), and stored at −80 °C until the analyses were performed. Blood glucose, aspartate aminotransferase (AST), alanine aminotransferase (ALT), total cholesterol (TC), high-density lipoprotein cholesterol (HDL-c), low density lipoprotein cholesterol (LDL-c), and triglycerides (TG) concentrations were determined by means of an autoanalyzer (Pentra C-200; HORIBA ABX, Madrid, Spain) following standardized procedures. Insulin, C-reactive protein (CRP), leptin, and adiponectin concentrations were determined using specific Enzyme-Linked ImmunoSorbent Assay (ELISA) kits (Demeditec; Kiel-Wellsee, Germany) in another autoanalyzer (Triturus; Grifols, Barcelona, Spain). Hepatic Steatosis Index (HSI) was calculated using the following formula [27]: (HSI) = 8 × (ALT/AST ratio) ± BMI (± 2, if female; ±2, if diabetes mellitus). HSI values < 30 rule out NAFLD with a sensitivity of 93.1%, while values > 36 detect NAFLD with a specificity of 92.4% [27]. Smoking status categorized participants as smokers or non-smokers. Those who reported as smoking at least sporadically during the last year were considered as smokers while those who reported as having stopped smoking at least one year before their enrolment in the study and those who had not smoked during their life were defined as non-smokers. The validated Spanish version of the Minnesota Leisure-Time Physical Activity Questionnaire was used to assess physical activity [28].

### 2.3. Liver Stiffness Assessment

Acoustic Radiation Force Impulse (ARFI) elastography (Siemens ACUSON S2000 and S3000) was the method used to assess liver stiffness [29]. The measurements were taken under fasting conditions and ARFI was carried out along with the ultrasonography when assessing liver steatosis. The median value of the 10 ARFI valid measurements that were performed in each patient was registered and used for further analyses [9].

### 2.4. Sleep Quality Assessment

Sleep characteristics (sleep duration, sleep efficiency, total time in bed, sleep disturbances and sleep quality) were assessed in both groups using the validated Spanish version of the Pittsburgh Sleep Quality Index (PSQI) as reported elsewhere [30]. This tool has shown a strong reliability and validity and has been used in a wide variety of samples [31,32,33]. The PSQI consists of 19 self-administered questions that generate 7 component scores with subscales ranging from 0 to 3. The sum of these components scores leads to a global score ranging from 0 to 21, for the assessment of sleep quality. A punctuation of more than 5 in the global score identifies “poor sleepers”, while a punctuation of 5 or lower identifies “good sleepers” [34]. Thus, poorer sleep quality is characterized by higher scores [30,35]. Short sleep duration was defined as a self-reported sleep time ≤ 6 h per night [15,22,36].

### 2.5. Dietary Assessment

The diet of the participants was assessed with a semiquantitative food frequency questionnaire (FFQ) of 137 items, previously validated in Spain for energy and nutrient intake. The nutrient composition of the food items was derived from accepted Spanish food composition tables [37,38].

### 2.6. Statistical Analyses

Variable distribution was assessed by means of the Shapiro-Wilk test. Data were presented as mean ± standard deviation or mean ± standard error. Groups were compared by the Student’s *t*-test for unpaired samples when data followed a normal distribution and the Mann-Whitney U test when data did not show a normal distribution. Comparison between groups was adjusted by BMI using Analysis of covariance (ANCOVA). Categorical variables were compared using the Chi-squared test. To evaluate the relationship between variables, the Pearson’s correlation coefficient or the Spearman’s rho were performed for parametric and non-parametric variables, respectively. Logistic regression models were set up to evaluate the risk of NAFLD (dependent variable) associated with sleep quality variables (independent variables). A linear regression analysis was performed to assess the influence of sleep characteristics in the variability of liver stiffness measured by ARFI. Multiple variable linear regression models were adjusted for potential confounders considering age, sex, physical activity, smoking status, and others when indicated. Software Stata version 12.0 (StataCorp, College Station, TX, USA) was used for the analyses. A *p*-value < 0.05 was considered statistically significant and all *p*-values presented were two-tailed.

## 3. Results

### 3.1. Characteristics of the Participants

A total of 94 patients with NAFLD and 40 normal weight controls were included in the study, whose main anthropometrical, clinical, and biochemical features are reported (Table 1). NAFLD patients were older than controls and the sex distribution was different between groups with more women in the control group than in the NAFLD group. Therefore, further analyses were adjusted by age and sex when required. Anthropometric and body composition measurements were significantly higher in NAFLD participants than in controls. Regarding lipid profile, HDL cholesterol was significantly lower, and triglycerides were significantly higher in NAFLD patients as compared to the control group. There were significant differences in glycemic profile (glucose, insulin, and HOMA-IR) and other metabolic parameters, such as leptin, ALT, AST, and ARFI values, in the NAFLD group compared to the control group. Adiponectin concentrations were significantly higher in the control group as compared to the NAFLD group.

Due to the differences in BMI between the two groups, an analysis adjusted by BMI was carried out to corroborate that the discrepancies observed in the parameters could be attributed to the presence or absence of hepatic steatosis (Table 1). After this analysis, only the differences in blood pressure, anthropometric measurements, leptin, adiponectin, and ALT were significant.

Regarding dietary intake and lifestyle factors (Table 2), there were no significant differences between groups in total energy consumption (*p* = 0.906) and macronutrient distribution (carbohydrates, *p* = 0.922; proteins, *p* = 0.212; lipids, *p* = 0.159). Nevertheless, the total ingestion of dietary fiber (*p* = 0.001) and vegetables (*p* < 0.001) were higher among the controls, while the intake of meat products (*p* = 0.019) was higher in the NAFLD group. Notably, physical activity (*p* < 0.001), was significantly lower in the NAFLD subjects, compared to the controls. Finally, there was no significant difference in smoking status (*p* = 0.080) between groups.

**Figure 1:**
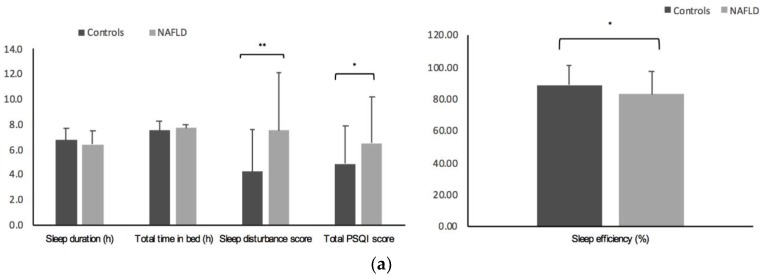

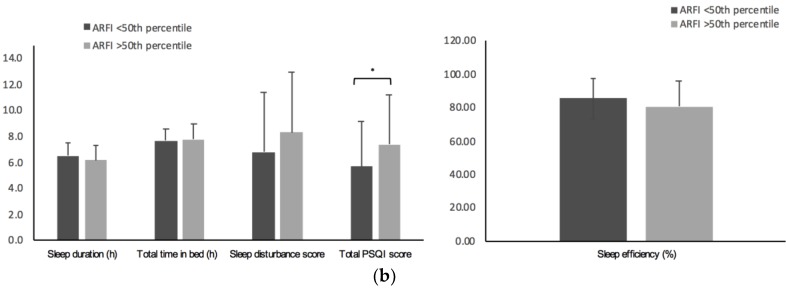
(**a**) Sleep characteristics of patients with NAFLD (*n* = 94) and normal weight controls (*n* = 40). (**b**) Sleep characteristics of patients with NAFLD and liver stiffness < 50th percentile (*n* = 46) vs. liver stiffness > 50th percentile (*n* = 46). Liver stiffness assessed by Acoustic Radiation Force Impulse (ARFI) elastography. Data expressed as mean ± SD CI95%, * *p* < 0.05, ** *p* < 0.01.

Sleep characteristics of patients with NAFLD compared to normal weight controls are shown (Figure 1a). When classifying sleep duration as ≤6 h or >6 h, short sleep duration was more prevalent in the NAFLD group than in controls (51% vs. 25%; *p* = 0.005). Moreover, sleep disturbance scores and sleep quality scores (total PSQI score) were significantly higher in patients with NAFLD, and when differentiating those with total PSQI scores of 5 or less (“good sleepers”) from those with scores > 5 (“poor sleepers”), poor sleep was more frequent in NAFLD patients than in controls (54% vs. 35%; *p* = 0.041). Sleep efficiency was significantly lower in the NAFLD group compared to the control group (*p* = 0.028). After the analysis was adjusted by BMI when the data were considered as continuous variables, there were no differences between groups.

When comparing ARFI elastography values within NAFLD patients (Figure 1b), those with higher values of liver stiffness (ARFI > 50th percentile) had a significantly higher total PSQI score compared with those with lower liver stiffness (ARFI < 50th percentile). Moreover, poor sleep quality was more common in the group with higher liver stiffness values than in the lower liver stiffness group (65% vs. 43%; *p* = 0.036). When comparing short sleep duration, the difference between higher and lower liver stiffness groups was not statistically significant (61% vs. 43%; *p =* 0.095).

### 3.2. Association between Sleep Characteristics and Risk of NAFLD

In the logistic regression analyses (Table 3), the age and sex adjusted OR (95% CI) for NAFLD when sleep duration > 6 h was 0.33 (0.13; 0.84) showing a protective effect (model 1). After multiple variable adjustment, including age, sex, physical activity, insulin, and smoking status, the odds ratio (95% confidence interval) for NAFLD was 0.15 (0.02; 1.04) and sleep duration was not significant (model 5). The association between NAFLD and the sleep disturbance score was 1.59 (1.11; 2.28) and remained significant after the multiple adjustment, suggesting that sleep disturbance may be a risk variable for hepatic steatosis (model 5).

### 3.3. Association between Sleep Characteristics and Liver Stiffness Assessed by ARFI

Linear regression analysis for ARFI was performed to assess the influence of sleep characteristics on liver stiffness (Table 4). After multiple adjustment for age, sex, smoking status, percentage of fat mass, physical activity, and total energy intake (model 4), the sleep disturbance score showed a positive association with liver stiffness (β 0.04 (95% IC 0.005; 0.07; *p* = 0.024)). In model 5, which considered the “coughing or snoring” item, significant associations between the sleep disturbance score and sleep quality (total PSQI score) with liver stiffness were found. In this model, variables considered together explained up to 20.3% of the variation of ARFI (adjusted-R^2^ = 0.203; *p* model = 0.001), when performed for the sleep disturbance score and up to 20.4% (adjusted-R^2^ = 0.204; *p* model = 0.001) when performed for sleep quality. In order to differentiate the influence of BMI, in model 6, the percentage of fat of model 5 was substituted by BMI and sleep variables were not significant.

### 3.4. Correlation of Sleep Characteristics and Variables Related to Hepatic Status (S1)

The relationship between sleep characteristics and the variables related to hepatic status in NAFLD patients and lean controls was separately analyzed in both groups. ARFI values were inversely correlated with sleep duration and sleep efficiency and positively associated to sleep disturbance and sleep quality (total PSQI score). These associations were only observed in the NAFLD group. When analyzing transaminases, no significant associations were found in the NAFLD group, while in the control group, ALT levels were inversely associated to sleep duration and AST concentration was positively associated to total PSQI score. The BMI was inversely associated to sleep duration and sleep efficiency in the NAFLD group. Android fat mass was also inversely associated with sleep duration in NAFLD subjects while no significant association was observed in the control group. Adiponectin concentration was positively associated with sleep duration in the NAFLD group and with the sleep disturbance score in the control group.

## 4. Discussion

There are well known risk factors for NAFLD, including obesity, type 2 diabetes mellitus, and metabolic syndrome [3]. However, this condition is influenced by multiple aspects, including genetic, demographic, clinical, and environmental determinants [39]. In this context, the evaluation of the possible influence of other putative factors is gaining relevance. Thus, in the present study, we analyzed the association between sleep disturbances and liver status in obese subjects with NAFLD compared with lean controls. We found that hepatic status, anthropometric measurements, and clinical and biochemical markers as well as some dietary components significantly differed in NAFLD subjects compared to the control group. Furthermore, sleep efficiency, sleep disturbance, and sleep quality differed between groups too. Moreover, a higher prevalence of short sleep duration and a poorer sleep quality were found among subjects with NAFLD. This significance was more robust in relation to short sleep duration than to sleep quality. Additionally, sleep disturbance was associated with a higher risk of NAFLD. Interestingly, sleep quality and sleep disturbance predicted the variability of liver stiffness after adjustment by potential confounders in NAFLD subjects.

Our results corroborate that blood pressure, anthropometric measurements, leptin, adiponectin, and ALT differ in NAFLD individuals compared to subjects without NAFLD, as found in previous studies [16,40,41], even when adjusting by BMI. Anyway, some of the differences between obese individuals with NAFLD and lean control subjects may be attributed to differences in BMI and not to their hepatic status. This study did not contemplate a group of obese subjects without NAFLD. However, obesity is closely related to NAFLD [42]. In an Italian population, an ultrasonographical NAFLD prevalence of 91% was reported in subjects with obesity (BMI ≥ 30 kg/m^2^), 67% in overweight individuals (BMI 25–30 kg/m^2^), and 25% in normal weight persons (BMI 18–25 kg/m^2^). Hence, prevalence of NAFLD rises with BMI [1]. Consequently, it is a more common scenario to find subjects with obesity and NAFLD than obese subjects without NAFLD.

To evaluate the differences in diet between both groups, we used a semiquantitative FFQ. Interestingly, there were no significant differences between groups in total energy and macronutrient consumption, but NAFLD subjects reported a lower intake of fiber and vegetables and a higher ingestion of meat products, while physical activity was higher in the control group. There are some factors that may explain the similar energy intake reported by both groups. First, the NAFLD patients were older than the control group, thus a decrease in energy requirements might be expected; second, the control group reported a significantly higher physical activity, therefore, this factor could contribute to higher energy requirements. Third, the underreporting of food intake may be more frequent in individuals with excessive body weight than in lean persons [43]. On the other hand, previous studies support that fiber may play an important role in liver diseases and that diverse liver damage markers are lower in subjects with higher fiber consumption [4,44]. Regarding meat ingestion, it has been described that patients with high Fatty Liver Index have a higher red meat intake [45] and that red meat may be associated with an increased risk of chronic liver disease [46]. Those findings suggest that not only energy intake, but also other dietary components and lifestyle factors have an influence on hepatic health.

Additionally, dietary intake has been proposed as a mediator of the association between short sleep duration, obesity and related comorbidities [18]. Thus, a relationship of short sleep duration with a higher total energy intake has been found [47,48] and a trend towards higher fat intake is suggested [47,49]. Therefore, this may contribute to an increased risk of obesity related chronic diseases [18]. However, we did not find significant correlations between sleep characteristics and dietary factors.

The PSQI is a widely used self-report questionnaire that evaluates subjective sleep quality, which has shown good reliability and validity for both healthy and clinical groups with diverse conditions, including mental and health problems, in different age groups and in a variety of cultural contexts [50]. In this study, sleep indicators, such as sleep duration, sleep efficiency, sleep disturbance, and sleep quality, were poorer in patients with NAFLD, but these differences could not be ascribed to NAFLD itself, thus BMI may play an important role in the interplay between sleep features and hepatic steatosis. In this context, previous research found that sleep duration was shortened and sleep quality was poor in patients with NAFLD [16]. However, other studies did not report significant differences in sleep duration between NAFLD subjects and healthy controls [51]. In addition, two recent systematic reviews and meta-analysis drew conflicting conclusions based on their evaluation. One of them found a small, but significant increase in the risk of NAFLD with short sleep duration [52], while the other found that short or long sleep duration was not significantly associated with the risk of fatty liver disease [53]. The lack of consensus in the definition of short sleep duration [18] and the different methods used to register sleep patterns that include subjective and objective measurements [54,55] may be in part responsible for inconsistencies in the results. Moreover, self-perceived sleep quality represents a challenge to define, to measure, and to classify because there is no generally accepted reference or gold standard for this construct [31]. In our sample, the analysis showed an increased risk of NAFLD in relation to sleep indicators, however, only the sleep disturbance score remained significant after multiple adjustment. It is important to mention that when adjusting sleep features for multiple variables in the logistic regression models, age, physical activity, and insulin were also significant. These results may indicate that sleep duration as well as other characteristics of sleep patterns should be specifically considered when evaluating NAFLD and when developing strategies for the treatment of this condition, along with the traditionally contemplated risk factors.

The biophysiological mechanisms underlying the association between short sleep duration and NAFLD are not completely understood, but there is compelling evidence that insufficient sleep promotes weight gain, obesity, insulin resistance, metabolic syndrome, and diabetes mellitus [56]. In addition, the reduction of leptin [57,58] and elevation of ghrelin [58] have been suggested as effects of sleep insufficiency. Thus, short sleep duration may be related to the distribution, timing, and behavior of the intake [48,58]. Furthermore, it has been suggested that insufficient sleep may predispose to NAFLD by means of proinflammatory markers and stress response [56].

Regarding liver stiffness, in patients with NAFLD, an association was found with short sleep duration, sleep efficiency, sleep disturbance, and overall sleep quality. In the multivariable analysis, the inclusion of coughing or snoring was considered as a confounding variable because it is associated with the risk of obstructive sleep apnea syndrome (OSAS) [59,60,61], which is the most studied sleep disorder and has been related to liver fibrosis [62,63,64]. Several studies have associated OSAS with hepatic inflammation and it is suggested that the chronic intermittent hypoxia in these individuals may play a role in the pathogenesis of NAFLD and in the progression from steatosis to steatohepatitis, cirrhosis, and hepatocellular carcinoma [42]. In our NAFLD sample, only three patients reported a previous diagnosis of OSAS, but it should be considered that OSAS affects 35–45% of individuals with obesity and that these two conditions often coexist and share common molecular mechanisms that lead to metabolic alterations [42]. Consequently, OSAS may be undiagnosed in a percentage of the individuals in our sample and sleep characteristics in NAFLD subjects are described in this study independent of the etiology. In addition, when the regression model considered BMI instead of the percentage of fat, the sleep variables were not significant. Nevertheless, fat percentage reflects body composition more accurately than BMI. However, in our analysis, the association of sleep disturbance and sleep quality was consistent and independent of “coughing or snoring”, but it is important to note that the prediction of the model was considerably raised when including this last variable. Therefore, these findings suggest that liver stiffness may be associated with different characteristics of sleep patterns besides only those derived from the risk of OSAS.

On the other hand, a previous study found an association between daytime sleepiness and the degree of liver fibrosis assessed by biopsies in NAFLD patients [16], being recognized that the risk of excessive daytime sleepiness is high among subjects with established liver cirrhosis [65,66]. In this case, the alteration of sleep may not be a cause, but a consequence of the hepatic disfunction. Likewise, daytime sleepiness is considered a symptom of OSAS [59]. However, in our study, daytime sleepiness was not significantly associated with liver stiffness (Table S1). These results may be explained by the diversity of methods used to assess liver fibrosis, which includes liver biopsies, ARFI elastography, transient elastography, among others. Furthermore, daytime sleepiness was registered using PSQI, which only includes one question regarding feelings of sleepiness in contrast to other instruments, such as the Epworth Sleepiness Scale, a frequently used instrument to auto-report the habitual chances of falling asleep in a variety of common situations [67].

There are some limitations in the present study that should be mentioned: Firstly, due to the cross-sectional design of the study, causal inferences cannot be made. Secondly, since matching of the age and sex of the control group with NAFLD subjects was a methodological difficulty, multivariable analysis, including age and sex as covariables, was performed to overcome this situation. Thirdly, sleep and dietary evaluations were carried out using self-reported information of the participants. Thus, subjective measures may produce some biases. Fourthly, the screening of participants, including information about competing causes of liver disease (endocrine disorders, infection with hepatitis virus, among others), was based on a clinical interview rather than a specific laboratory assessment and the diagnosis and evaluation of NAFLD was based on imaging techniques rather than histological methods. Fifthly, we could not perform a diagnostic evaluation of OSAS in our patients. Sixthly, this study included a lean control group without NAFLD and an obese group with NAFLD. A methodological drawback should be noted, since the control group is non-obese. Additionally, this study did not include a group matched for BMI without NAFLD. Therefore, it could not be confirmed that sleep disturbances were due exclusively to NAFLD and not to the excessive body weight.

To our knowledge, few studies have detailed this variety of sleep features in subjects with NAFLD and analyzed the relationship of these variables with ARFI assessed liver stiffness. Based on the findings reported here, further studies should analyze more comprehensively the association between sleep characteristics and NAFLD, not only taking into consideration sleep duration, but other sleep features, such as sleep disturbance, sleep efficiency and overall sleep quality, among others, which may have impact on the development and progression of NAFLD. Moreover, interventional strategies focused on sleep behavior changes may be contemplated in subjects with NAFLD. Additionally, to examine the consistency of the results, further studies should compare data measured by objective (actigraphy, polysomnography, etc.) and subjective sleep parameters in patients with NAFLD and differentiate those subjects with OSAS from those without OSAS because general disagreements in the associations may depend on the method used for the sleep evaluation [68,69] and situation, which may contribute to explain the discrepancies reported in the literature. Furthermore, subjects should be better characterized, taking into consideration other emerging conditions that are also associated with NAFLD, such as hypothyroidism [70] and other endocrinopathies, osteoporosis, colorectal cancer, and psoriasis [3]. Finally, since the liver plays an important role in the regulation of hormones, such as melatonin, sleep disturbances could be evaluated from the perspective of the consequences of NAFLD.

## 5. Conclusions

This data supports the association of sleep characteristics with the development and progression of NAFLD. Sleep disruption may be contributing to the pathogenesis of NAFLD as well as the alteration of the liver may be affecting sleep parameters. These findings suggest that sleep quality and related factors may be added to the list of modifiable behaviors to consider in health promotion strategies and in the prevention and management of NAFLD in diverse clinical settings.

## Figures and Tables

**Table 1:** Anthropometry, clinical features, and biochemical parameters of participants with ultrasound diagnosed NAFLD (*n* = 94) and controls (*n* = 40).

	Controls Mean ± SD	NAFLD Mean ± SD	*p*	Controls ^#^ Mean ± SE	NAFLD ^#^ Mean ± SE	*p* ^#^
Age (years)	41.5 ± 9.8	51.4 ± 8.9	<0.001	39.4 ± 9.8	51.4 ± 8.9	<0.001
Weight (kg)	62.1 ± 10.0	94.6 ± 13.6	<0.001	-	-	-
BMI (kg/m^2^)	22.1 ± 1.8	33.7 ± 3.9	<0.001	-	-	-
Sex (men/women)	14/26	53/41	0.023	-	-	-
SBP (mmHg)	108 ± 17	132 ± 15	<0.001	114 ± 4	129 ± 2	0.005
DBP (mmHg)	69 ± 10	87 ± 9	<0.001	72.8 ± 2.3	85.3 ± 8	<0.001
Waist circumference (cm)	75.6 ± 7.3	109.0 ± 8.8	<0.001	90.7 ± 1.5	102.7 ± 0.8	<0.001
DXA Total adipose tissue (%)	26.8 ± 7.6	42.3 ± 6.5	<0.001	33.4 ± 1.7	39.6 ± 0.9	0.006
DXA Visceral fat mass (kg)	0.2 ± 0.21	2.3 ± 1.08	<0.001	0.3 ± 0.25	2.3 ± 0.13	<0.001
Total cholesterol (mg/dL)	191.5 ± 30.6	192.4 ± 39.5	0.906	183.7 ± 9.7	196.2 ± 5.0	0.344
HDL cholesterol (mg/dL)	63.1 ± 11.7	51.8 ± 14.3	<0.001	56.5 ± 3.5	54.5 ± 1.8	0.682
LDL cholesterol (mg/dL)	114.7 ± 26.9	113.8 ± 35.7	0.896	114.7 ± 26.9	113.8 ± 35.7	0.607
Triglycerides (mg/dL)	68.8 ± 40.4	135.4 ± 77.9	<0.001	86.0 ± 18.1	128.1 ± 9.4	0.086
Fasting glucose (mg/dL)	85.4 ± 6.6	106.4 ± 31.1	<0.001	104.3 ± 6.6	98.5 ± 3.5	0.511
Insulin (mU/L)	4.3 ± 2.0	18.6 ± 10.7	<0.001	12.2 ± 2.2	15.3 ± 1.1	0.312
HOMA-IR	0.9 ± 0.5	5.1 ± 4.8	<0.001	4.7 ± 1.5	3.6 ± 0.5	0.416
Leptin (ng/mL)	10.0 ± 8.0	40.1 ± 33.5	<0.001	43.7 ± 6.5	25.7 ± 3.4	0.043
Adiponectin (µg/mL)	13.5 ± 4.7	6.8 ± 2.3	<0.001	11.9 ± 0.8	7.5 ± 4.2	<0.001
C-reactive protein (mg/dL)	0.47 ± 0.6	0.45 ± 0.6	0.853	0.72 ± 0.2	0.34 ± 0.1	0.061
AST (IU/L)	21.4 ± 6.4	24.5 ± 9.9	0.035	19.9 ± 2.4	25.3 ± 1.2	0.094
ALT (IU/L)	17.2 ± 13.5	33.7 ± 18.2	<0.001	17.9 ± 4.5	33.5 ± 2.3	0.010
Hepatic Steatosis Index (HSI)	29.6 ± 3.1	45.4 ± 45.4	<0.001	-	-	-
ARFI liver stiffness (m/s)	1.34 ± 0.2	1.86 ± 0.7	<0.001	1.82 ± 0.1	1.65 ± 0.8	0.396

Nonalcoholic fatty liver disease (NAFLD); Body Mass Index (BMI); Systolic blood pressure (SBP); Diastolic blood pressure (DBP); Homeostasis model assessment of insulin resistance (HOMA-IR); Asparate aminotransferase (AST); Alanine aminotransferase (ALT); Acoustic radiation force impulse (ARFI); ^#^ Adjusted by BMI.

**Table 2:** Daily nutrient intake and lifestyle factors of participants with ultrasound diagnosed NAFLD (*n* = 94) and controls (*n* = 40).

	Controls Mean ± SD	NAFLD Mean ± SD	*p*
Energy and macronutrients			
Total energy (kcal)	2677 ± 749	2697 ± 1089	0.906
Carbohydrates (%)	43 ± 6.6	43 ± 7.0	0.922
Proteins (%)	16 ± 3.2	17 ± 3.8	0.212
Lipids (%)	39 ± 5.5	37 ± 7.0	0.159
Dietary fiber (g)	33 ± 16	25 ± 9	0.001
Food groups			
Fruit (g)	345 ± 179	290 ± 197	0.141
Vegetables (g)	431 ± 236	285 ± 120	<0.001
Legumes (g)	21 ± 14	21 ± 10	0.738
Fish (g)	99 ± 46	88 ± 45	0.232
Meat products (g)	154 ± 80	190 ± 78	0.019
Micronutrients			
Vitamin A (µg)	1526 ± 650	1119 ± 893	0.014
Vitamin C (mg)	250 ± 76	192 ± 98	0.001
Vitamin D (µg)	8 ± 3.8	6 ± 4.0	0.041
Vitamin E (mg)	12 ± 4.1	10 ± 4.3	0.017
Vitamin B9 (µg)	445 ± 132	360 ± 151	0.003
Marine Omega-3 (g)	0.90 ± 0.5	0.62 ± 0.5	0.003
Lifestyle factors			
Physical Activity (METs-min/week)	5801 ± 4225	3049 ± 2440	<0.001
Smokers (%)	35.0	20.7	0.080

**Table 3:** Association between sleep characteristics and risk for hepatic steatosis assessed by ultrasonography in NAFLD patients (*n* = 94) and controls (*n* = 40).

	Model 1	Model 2	Model 3	Model 4	Model 5
	OR (95% CI)	OR (95% CI)	OR (95% CI)	OR (95% CI)	OR (95% CI)
Sleep duration ≤6 h or >6 h	0.33 (0.13; 0.84) *	0.37 (0.14; 0.98) *	0.39 (0.09; 1.71)	0.34 (0.13; 0.85) *	0.15 (0.02; 1.04)
Sleep efficiency (%)	0.974 (0.94; 1.009)	0.981 (0.94; 1.01)	0.979 (0.93; 1.02)	0.975 (0.94; 1.01)	0.981 (0.93; 1.03)
Total time in bed (h)	1.19 (0.75; 1.88)	1.11 (0.67; 1.83)	1.79 (0.75; 4.29)	1.20 (0.76; 1.89)	1.29 (0.50; 3.32)
Sleep disturbance score	1.23 (1.08; 1.39) **	1.21 (1.06; 1.38) **	1.38 (1.08; 1.75) **	1.22 (1.08; 1.39) **	1.59 (1.11; 2.28) *
Sleep quality (Total PSQI score)	1.15 (1.01; 1.33) *	1.13 (0.98; 1.31)	1.14 (0.93; 1.39)	1.15 (0.99; 1.32)	1.10 (0.88; 1.38)

Odds Ratio (95% confidence interval) for hepatic steatosis were compared by logistic regression. *Model 1*: adjusted for age and sex. *Model 2*: adjusted for age, sex and physical activity (METs). *Model 3*: adjusted for age, sex and insulin. *Model 4*: adjusted for age, sex and smoking. *Model 5*: adjusted for age, sex, physical activity (METs), insulin and smoking. * *p* < 0.05, ** *p* < 0.01.

**Table 4:** Regression analysis of sleep characteristics and liver stiffness assessed by ARFI in patients with NAFLD.

		β (95% IC)	*p*	Adjusted *R*^2^	*p* Model
Sleep duration ≤6 h or >6 h	Model 1	−0.30 (−0.57; −0.02)	0.034		
	Model 2	−0.28 (−0.56; −0.002)	0.048	0.028	0.135
	Model 3	−0.25 (−0.54; 0.02)	0.078	0.099	0.022
	Model 4	−0.22 (−0.53; 0.08)	0.154	0.058	0.123
	Model 5	−0.27 (−0.55; 0.01)	0.059	0.188	0.002
	Model 6	−0.22 (−0.50; 0.06)	0.114	0.205	<0.001
Sleep Efficiency	Model 1	−0.01 (−0.02; −0.0002)	0.045		
	Model 2	−0.009 (−0.01; 0.0005)	0.063	0.023	0.165
	Model 3	−0.008 (−0.01; 0.001)	0.087	0.097	0.023
	Model 4	−0.008 (−0.02; 0.002)	0.131	0.061	0.114
	Model 5	0.008 (−0.01; 0.0008)	0.075	0.184	0.002
	Model 6	−0.005 (−0.01; 0.004)	0.248	0.195	0.001
Total time in bed	Model 1	0.02 (−0.11; 0.15)	0.750		
	Model 2	0.01 (−0.12; 0.15)	0.805	−0.014	0.646
	Model 3	0.002 (−0.13; 0.13)	0.974	0.064	0.072
	Model 4	−0.01 (−0.16; 0.13)	0.863	0.031	0.233
	Model 5	−0.01 (−0.14; 0.11)	0.868	0.150	0.007
	Model 6	−0.02 (−0.14; 0.10)	0.781	0.182	0.002
Sleep disturbance score	Model 1	0.03 (0.001; 0.06)	0.037		
	Model 2	0.02 (−0.001; 0.06)	0.064	0.023	0.166
	Model 3	0.02 (−0.002; 0.05)	0.069	0.102	0.020
	Model 4	0.04 (0.005; 0.07)	0.024	0.097	0.042
	Model 5	0.04 (0.005; 0.07)	0.024	0.203	0.001
	Model 6	0.03 (0.004; 0.07)	0.081	0.212	<0.001
Sleep quality (Total PSQI score)	Model 1	0.04 (0.004; 0.07)	0.029		
	Model 2	0.03 (−0.0002; 0.07)	0.051	0.027	0.142
	Model 3	0.04 (0.006; 0.08)	0.022	0.123	0.009
	Model 4	0.04 (0.002; 0.09)	0.039	0.086	0.057
	Model 5	0.04 (0.006; 0.08)	0.023	0.204	0.001
	Model 6	0.03 (−0.005; 0.07)	0.085	0.211	<0.001

Model 1: unadjusted variable. Model 2: adjusted for age and sex. Model 3: adjusted for age, sex, smoking, fat mass (%), physical activity (METs). Model 4: adjusted for age, sex, smoking, fat mass (%), physical activity (METs) and total energy intake (kcal/day). Model 5: adjusted for age, sex, smoking, fat mass (%), physical activity (METs) and coughing or snoring. Model 6: adjusted for age, sex, smoking, BMI, physical activity (METs) and coughing or snoring.

## Supplementary Materials

The following are available online at http://www.mdpi.com/2072-6643/11/2/322/s1, Table S1: Correlations of sleep characteristics and hepatic status related variables of patients with nonalcoholic fatty liver disease and controls.
