# Supplementary material for: Association between Sleep Disturbances and Liver Status in Obese Subjects with Nonalcoholic Fatty Liver Disease: A Comparison with Healthy Controls
Source: Nutrients. 2019 Feb 2;11(2):322. doi: 10.3390/nu11020322 (PMC6412197; doi:10.3390/nu11020322)
Supplement: Supplementary file 1 [file nutrients-11-00322-s001.pdf]

Table S1. Correlations of sleep characteristics and hepatic status related variables of patients with nonalcoholic fatty liver disease and controls.

	ARFI (m/s)	Steatosis stage	AST (UI/L)	ALT (UI/L)	BMI (kg/m^2)	Leptin (ng/mL)	Adiponectin (µg/mL)
**NAFLD (n=94)**							
Sleep duration (h)	*r*= -0.215	*r*= -0.188	*r*=-0.089	*r*=-0.043	*r*= -0.325	*r*=-0.186	*r*= 0.230
	p= 0.039	*p*=0.069	*p*=0.392	*p*=0.677	*p*=0.001	*p*=0.072	*p*= 0.025
Sleep efficiency (%)	*r*= -0.169	*r*= -0.259	*r*= -0.041	*r*=-0.094	*r*= -0.204	*r*=-0.120	*r*= 0.121
	p=0.107	*p*= 0.011	*p*=0.690	*p*=0.364	*p*=0.048	*p*=0.248	*p*= 0.245
Total time in bed (h)	*r*= 0.036	*r*=0.092	*r*=0.113	*r*=0.016	*r*= -0.063	*r*= -0.023	*r*=-0.057
	p=0.730	*p*=0.376	*p*=0.278	*p*=0.877	*p*=0.542	*p*=0.821	*p*=0.582
Daytime sleepiness	*r*= 0.065	*r*=0.154	*r*=-0.004	*r*=0.068	*r*= 0.154	*r*= 0.045	*r*= 0.008
	p=0.537	*p*=0.138	*p*=0.969	*p*=0.517	*p*=0.138	*p*=0.665	*p*=0.935
Sleep disturbance score	*r*= 0.240	*r*= *0.*051	*r*= -0.033	*r*= 0.077	*r*= 0.123	*r*= 0.177	*r*= *0.195*
	p= *0.020*	*p*= *0.624*	*p*= *0.749*	*p*= *0.456*	*p*= 0.235	*p*= *0.087*	*p*= *0.059*
Total PSQI score	*r*=0.257	*r*=0.144	*r*=0.015	*r*=0.119	*r*= 0.134	*r*=0.141	*r*=-0.109
	p=0.013	*p*=0.164	*p*=0.883	*p*=0.251	*p*= 0.197	*p*=0.172	*p*=0.837
**Controls (n=40)**							
Sleep duration (h)	*r*= 0.187	*r*= -	*r*= -0.258	*r*= -0.328	*r*= 0.004	*r*= 0.243	*r*= 0.024
	p= 0.247	*p*= -	*p*=0.107	*p*=0.038	*p*=0.977	*p*= 0.130	*p*= 0.881
Sleep efficiency (%)	*r*= 0.096	*r*= -	*r*= -0.140	*r*= -0.086	*r*= -0.023	*r*= 0.141	*r*= 0.074
	p=0.552	*p*= -	*p*=0.386	*p*=0.593	*p*= 0.884	*p*= 0.385	*p*= 0.650
Total time in bed (h)	*r*= 0.132	*r*= -	*r*= -0.302	*r*= -0.308	*r*= -0.016	*r*= 0.261	*r*= 0.192
	p=0.413	*p*= -	*p*= 0.058	*p*=0.052	*p*=0.919	*p*= 0.102	*p*= 0.235
Daytime sleepiness	*r*= 0.082	*r*= -	*r*=-0.042	*r*=-0.164	*r*= -0.171	*r*= -0.017	*r*= -0.100
	p=0.612	*p*= -	*p*=0.794	*p*=0.310	*p*=0.289	*p*=0.913	*p*=0.537
Sleep disturbance score	*r*= 0.010	*r*= -	*r*= 0.058	*r*= -0.124	*r*= -0.258	*r*= 0.139	*r*= *0.316*
	p= *0.947*	*p*= -	*p*= *0.719*	*p*= *0.445*	*p*= 0.106	*p*= *0.391*	*p*= *0.046*
Total PSQI score	*r*= -0.033	*r*= -	*r*= 0.324	*r*= 0.156	*r*= -0.243	*r*= -0.040	*r*= 0.172
	p= 0.839	*p*= -	*p*= 0.040	*p*= 0.335	*p*= 0.129	*p*=0.804	*p*= 0.288
